# The Book Wonderful

**Published:** 1881-04

**Authors:** Ausburn Towner


### THE BOOK WONDERFUL.

AUSBURN TOWNER.

The revision of the Bible, an undertaking that has been in the hands of a number of scholars for the past few years, the result of whose labors, already known in isolated parts and detached portions, will soon be given in full to the Christian world, makes of present interest some account of this remarkable book in its English dress. Whence its original source, its vitality and its enormous circulation, need no comments. How it appeared in our vernacular is a subject the details of which are little known, so little known as to make them novel and curious. It is only in connection with this modern revision of the book that inquiries have been led in this direction.

The very first translation into Anglo-Saxon of any portion of the Bible, that is now known of, was made about 690 A. D. by a monk named Cædmor, who has been called the father of English poetry. He translated into verses the narrative of the Creation, the Exodus, and the Incarnation and Passion of our Lord. It was not a literal translation except in a few of the sentences. Although this was twelve hundred years ago, copies of the work, of course in manuscript, are still in existence. Another ecclesiastic named Guthlac, translated the "Psalms" into Anglo-Saxon a few years later. Aldhelm, a Bishop, in 706 made another metrical translation of the Psalms.

The next translation made was of the Gospel of St. John, and it was done by the Venerable Bede. It is said that the work was completed on May 26, 745, the same day that the eminent man died. The Venerable Bede is one of the strongest and most prominent characters in English history and the race is indebted to him for all that is known of the period in which he lived and for many years preceding his time. He is said to have translated the Lord's Prayer, some portions of the Psalms and other parts of the Bible, but nothing remains to show that he did.

Alfred the Great put at the head of his well-known Code of Laws the Ten Commandments and other Mosaic injunctions, all in the Anglo-Saxon tongue. He was very desirous that all of his subjects should be able to read the Bible in their tongue. He began a translation of the Psalms, but died before he had completed it. It was in the seventh century also, that a priest wrote an interlinear translation in the Anglo-Saxon, of the Gospels in a beautiful Latin copy of them, and soon after a similar work was done by another monk. Three hundred years passed and then an Anglo-Saxon scholar named Ælfric, who afterward became Archbishop of York, translated a number of the books of the Old Testament. About 1100 an author named Orme made a paraphrase of the Gospels and Acts of the Apostles in blank verse.

These are about all of the translations of the Bible or portions of it, with the exceptions of the Psalter and parts of books, that are known to have been made into our mother tongue previous to 1380. Up to that year, the Bible was an unknown book to the mass of the English people, except as to its general character, but in that year, the immortal John Wycliffe completed his translation of the New Testament. By his preaching he had paved the way for a demand for the work and when it was finished he was unable to supply it. Every copy had to be written by hand, and it is said that some of the yeomen were so anxious to get one that they often gave a load of hay for a few chapters.

Wycliffe completed the translation of the Old Testament in 1384, and soon after he died. His translation was the first translation of the whole Bible into English as we have it now, that was ever made. It is difficult to understand the people of that day. It would not be accurate to call them fools, yet they acted very like such creatures. It was forty-three years after Wycliffe's death that, by a decree of the Roman Catholic Council of Constance, his body was dug up from the cemetery where it had been buried, was burned on an arch of a river near at hand and the ashes cast into the water. This river empties into the Avon and the idiotic transaction gave rise to the quaint verse:

The Avon to the Severn runs,
The Severn to the sea,
And Wycliffe's bones shall spread abroad,
Wide as the waters be.

This was in reference not only to Wycliffe materially, but to his work aud influence in translating the Bible.

Although every copy had to be written by hand, there must have been many of them transcribed, as there are nearly two hundred in existence, some of them having been the property of those who are called exalted personages. Henry VI., Richard III., Henry VII., Edward VI., Queen Elizabeth and Bishop Bonner, each owned a copy of the work.

Different portions of the Bible were also translated by other persons at this same period, but no complete translation had ever before been made.

The next English Bible was a very famous one and became known as Tyndale's Bible. Its translation was the work of William Tyndale, who devoted his life to it. About 1524 he published in Hamburg the first part of the Bible ever printed in the English language. It comprised the Gospels of Matthew and Mark. In about a year afterward, the first complete copy of the New Testament in English, was printed by him in Worms, where he had been driven by the persecutions of Cardinal Wolsey and other Englishmen. Three thousand copies were printed, and they met with a ready sale, for Wolsey had book agents who bought them up as fast as they could find them, and they were burned in the presence of the Cardinal and his clergy in front of St. Paul's Cathedral, London, on Sunday, February 11, 1526! One can hardly keep from laughing at such an absurd performance. The result was that Tyndale kept on printing his Bibles and realized so handsomely on their sale that he paid all of his debts, revised his translation and in due time issued a far larger and more accurate edition. However, before he had completed his translation of the Old Testament, they caught him, and he had the same fate meted out to him that had befallen some of his Bibles—he was burned.

He had left a friend behind him, however, named John Rogers, who went on with his work and only in the next year after his death, having assumed the name of Matthews, published a complete English version of the Bible, and this too, by royal authority.

Two years before this, an English Bible bearing the name of Miles Coverdale, and so, known as the Coverdale Bible, printed in Zurich, had an extensive circulation in England. It did not have the royal authority. Portions of it were incorporated into the Matthews Bible, above mentioned.

Four years after the publication of the Coverdale Bible, what is known as the Great Bible was issued. This was published at the instance of the clergy of England, who were not satisfied with any of the Bibles thus far printed. The Matthews Bible was the basis of this work, although they had recourse to the tongues in which it was originally written. The Book of Psalms was particularly revised and corrected, the translation having reference to the metrical and musical construction of the sentences so far as could be done without destroying their original meanings. The Psalms as printed in this edition of the Bible are those used in the Liturgy of the Episcopal church, and by comparison it will be seen that they differ somewhat from those in the Bibles in common use. The publication of this Great Bible was begun in Paris, but the Jesuits made the printers a great deal of trouble and the publisher took his workmen, presses, type and paper to London, where the book was issued in April, 1539, it being the first Bible printed by authority in England.

Something may be judged of the demand, the actual craving, it might be called, for this book in England, by the fact that from the time of the printing of Tyndale's New Testament in 1525 to 1542, when the Romish power got a little strength, there were issued thirty-nine editions of the New Testament and fourteen editions of the whole Bible, and that in the six short years of the reign of Edward VI., who succeeded Henry VIII. in 1547, no less than thirty-five editions of the New Testament and fifteen of the entire Bible were printed!

It is almost needless to say, that in the next five years, during the reign of Mary, not a Testament or Bible was printed in England.

What is known as the "Geneva Bible" was the next English version of the Scriptures printed. It gets its name from the city where it was published where its translator or revisor, William Whittingham, a noble Englishman, had been driven by persecutions at home. He issued his New Testament on June 10, 1557. It was the first Testament in which the text was divided into separate verses. This was following the example of Robert Stephen, another exiled and scholarly Englishman, who in 1551 printed his famous Greek Testament side by side with the Latin one in common use among the clergy.

To this Testament of Whittingham we are also indebted for a peculiarity that is annoying even to the best of readers, the use of italics for words which had no equivalents in the original, but which were added to complete the sense. It is to be supposed this method was adopted to show that the translator did not wish to deceive the readers in the least.

This Genevan Bible was the first one that was printed in the Roman style of type, the style in which the BISTOURY is printed. All the others were printed in "black letter," something like this: **Black Letter.** It was also, as has been said, the first Bible that had its text divided into verses.

The place of the origin of the Bible, Geneva, the home of Calvin, endeared it to the Puritans of England and the Presbyterians of Scotland, but it hardly suited the Established church. This body, therefore, under the lead of Archbishop Parker, soon after the accession of Queen Elizabeth, made a further revision to make it fully, as they said, represent the advances already made in Biblical literature. The revision was begun in 1564, and the publication was made in 1568, the work being known as "The Bishop's Bible." It was very defective in many respects, but it formed the basis of what is known as the "King James Bible" or the "Authorized version of the Scriptures," that was published in 1611. In this, forty-seven of the most eminent scholars of Great Britain were engaged. The manner in which the work was done and the extreme care taken, forms very entertaining reading for those at all interested in Biblical or literary subjects.

As showing the extreme care and caution used, it is to be observed that nearly three hundred years have elapsed and we are still using the same version that was made in the time of King James I. of England. To be sure, taking up the latest issue of the book and comparing it with one printed in 1611 and some differences would be observed; words printed in italics are changed; marginal readings have been added; old forms of words and occasionally expressions have been modernized, but all these changes would escape the notice of the great body of Christians and would be perceivable only to the most lerned and critical.

There are many who regard with excusable apprehension the publication of the new revision. The book we have had in use for nearly three centuries may have blemishes, too many perhaps, but they change no fact, alter no precept, obscure no doctrine; they may slightly mar the surface, but they do not destroy the exquisite symmetry nor touch the foundation of revealed truth; to the eye of the critic or one seeking faults, a word may be out of place or a human corruption may be here and there rudely inserted, but the Divine Word is there in all its substantial integrity. It remains to be seen whether or not the Christian religion is strong enough to stand unmoved when its most potent appeal to the mind and intelligence is in the least changed from what for three centuries has been esteemed to be its most accurate expression.

